# In Vitro and In Vivo Validation of Endothelium-Derived Potential Therapeutics for Myocardial Ischemia/Reperfusion Injury Identified by an AI-Enhanced Single-Cell and Virtual-Cell Paradigm

**DOI:** 10.3390/ijms27062743

**Published:** 2026-03-18

**Authors:** Qianlong Zhang, Yongsheng Liu, Zhichao Zhao, Yonggang Cao, Hongli Sun, Jianfa Wang, Rui Wu

**Affiliations:** 1 College of Animal Science and Veterinary Medicine, Heilongjiang Bayi Agricultural University, Daqing 163319, China; zhangqianlong@hmudq.edu.cn; 2 Quality Supervision, Inspection and Testing Center for Agricultural Processing Products of the Ministry of Agriculture, Daqing 163319, China; 3 College of Basic Medicine, Harbin Medical University-Daqing, Daqing 163319, China; liuyongsheng@hmudq.edu.cn (Y.L.); zzc-yjs2024022138@hmudq.edu.cn (Z.Z.); caoyonggang@hmudq.edu.cn (Y.C.); sunhongli@hmudq.edu.cn (H.S.); 4 College of Basic Medicine, Jiamusi University, Jiamusi 154007, China

**Keywords:** single-cell sequencing, endothelial cells, S100A8, andrographolide, inflammation

## Abstract

Myocardial ischemia/reperfusion (MI/R) injury affects heart attack outcomes. Endothelial cells dysfunction immediately after MI/R, but the key molecules and how to block them remain unclear. We combined single-cell atlas analysis, AI simulation, and experimental single-cell RNA sequencing data from mouse MI/R; we did quality control, cell annotation, hdWGCNA, and differential gene screening to identify endothelial genes. We constructed a protein network with STRING, predicted structure with AlphaFold3, and used AutoDock for molecular docking to find potential drugs. Virtual knockout simulations were used to check gene deletion effects. The compound andrographolide (AG) was tested in in vitro and in vivo MI/R models by measuring cell viability, inflammation, pathway activity, infarct size, and cardiac function. Single-cell analysis showed that S100 calcium binding protein A8 (S100A8) is an important element in vascular inflammation. It promotes inflammation by interacting indirectly with Cluster of differentiation 14 (CD14). Molecular docking showed that AG binds stably to S100A8. In vitro, AG reduced endothelial injury and blocked the IL-17 pathway. In vivo, AG reduced infarct size, improved cardiac function, and lowered S100A8 and IL-17 pathway proteins. Using single-cell analysis, AI, and experiments, we showed that S100A8 is related to MI/R injury. Andrographolide protects microvasculature via the S100A8 pathway, offering a promising treatment approach and new insights into heart injury mechanisms.

## 1. Introduction

Reperfusion therapy for restoring coronary blood flow after acute myocardial infarction (AMI) is a major part of modern cardiology to save lives. However, reperfusion itself causes further damage to the heart muscle; this is called ischemia/reperfusion (I/R) injury [1]. It severely limits the ultimate effectiveness of reperfusion therapy, and it is one of the main causes of adverse ventricular remodeling and heart failure [2]. Many potential treatment targets were identified in early studies; however, the majority of these have encountered significant challenges during clinical translation. This translational gap highlights the fundamental deficiency in how we understand Myocardial ischemia/reperfusion (MI/R) injury, particularly its early initiation and precise regulatory mechanisms. In the complicated pathologic web of MI/R injury, it has been proven that the dysfunction of the vascular endothelium is an important link [3]. Endothelial cells are the dynamic interface between the vascular lumen and myocardial tissue; they directly experience oxidative stress, calcium overload, and altered shear stress when blood flow is restored. They rapidly undergo phenotypic switching from an anti-inflammatory, anti-coagulant state to a state causing swelling and encouraging sticking [4]. By observing the increase in adhesion molecules (Intercellular adhesion molecule 1 (ICAM-1), Vascular cell adhesion molecule 1 (VCAM-1)) and the release of numerous chemokines (Interleukin 8 (IL-8), Monocyte Chemoattractant protein 1 (MCP-1)) [5,6], we can see that these result in the adhesion, infiltration, and amplification of parenchymal injury caused by inflammatory cells such as neutrophils in the microvasculature. Thus, we need to know the specific molecular mechanism of this early kind of switching in the endothelial cell so that we can do a proper intervention.

Due to the development of high-throughput sequencing technology, there are new ways to explore this problem from a system point of view. scRNA-seq can show the whole transcription scene and the differences between every type of cell in an injured myocardium with never-before-seen accuracy [7].

This technology is used in MI/R injury studies to delineate heterogeneous populations of cardiac endothelial cells and their differential responses to ischemic insult. It may also help us find the specific signaling pathways and transcriptional networks that are activated immediately following the start of tissue damage. This single-cell resolution shifts the paradigm from viewing endothelial cells as a homogeneous population to interrogating the specific cellular subsets that contribute to cardiac dysfunction, as visualized through transcriptomic profiling [8]. After obtaining key molecular candidates at the single-cell level, the research needs to delve further into the microscopic world of protein structure and function. Artificial intelligence is improving due to deep learning tools, such as AlphaFold3, which are changing this field. They have reached a new level of accuracy in predicting how proteins and groups of proteins will appear in 3D space [9]; they demonstrate strong agreement with experimentally determined structures in benchmark studies. Additionally, techniques such as molecular docking enable computer analyses to investigate why some proteins stick together while others do not.

This study adopts the “target discovery through single-cell atlas and AI-driven design” approach. The primary objectives of this study were twofold: First, to elucidate the dynamic functional transitions of cardiac endothelial cells—from their initial dysfunction during ischemia to their recovery phase upon reperfusion. Second, to find appropriate ways to help people after understanding these cellular responses. We utilized scRNA-seq to generate a dynamic picture of how heart cells converse with each other post-MI/R, concentrating on clusters of blood vessel cells to determine which ones immediately appear to battle an infection [10]. AI screening [11] provided us with a list of potential compounds that were subsequently tested on animals at various functional levels. It will provide new system biology insights into the endothelial aspect of MI/R damage, possibly providing robust experimental proof and prospective therapeutic medicines to assist treatment plans based on novel targets identified by this research.

## 2. Results

### 2.1. A Detailed Engineering Workflow Diagram Is Provided in Figure 1

**Figure 1:**
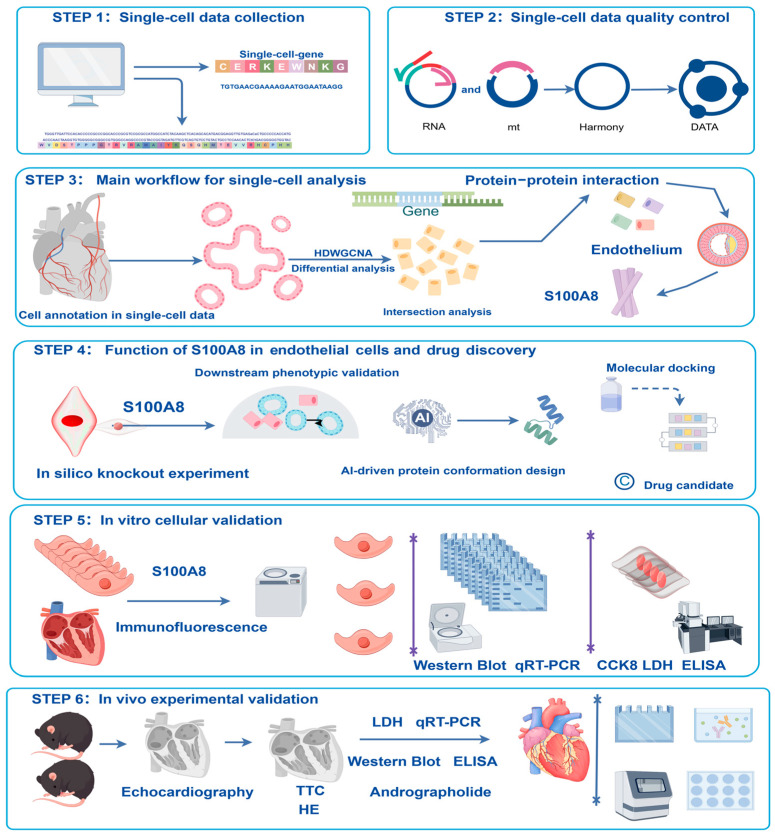
Flowchart showing the main methodology and technical procedures followed at each stage of the research.

The overall process of our experiment mainly involved first collecting a single-cell atlas and then conducting a series of analyses on the single-cell RNA sequencing data. Simultaneously, we used STRING to construct a protein network, employed AlphaFold3 to calculate protein–protein interactions, and utilized AutoDock for molecular docking to search for potential drugs. The impact of gene perturbations was examined through virtual knockout simulations. Finally, AG was administered to evaluate its therapeutic effect in in vitro and in vivo MI/R models (Figure 1).

### 2.2. Integration and Quality Control of Single-Cell RNA-Seq Data

**Figure 2:**
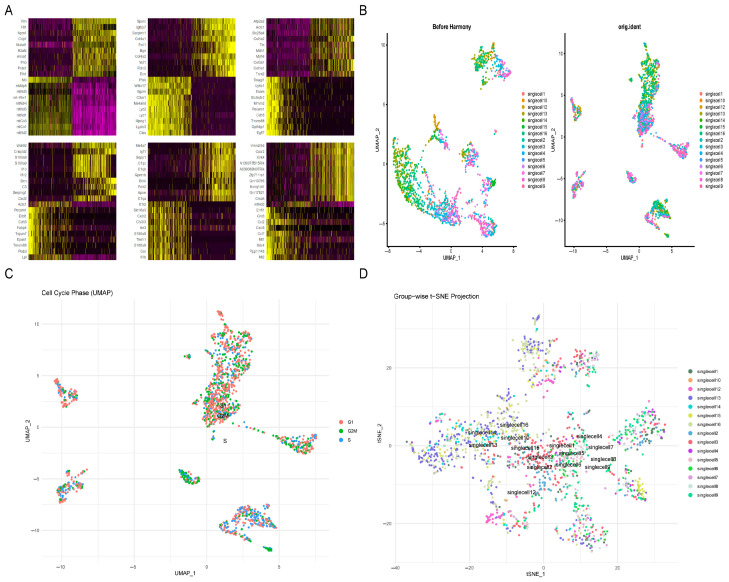
(**A**) Heatmap of feature gene expression from PCA. It displays the expression patterns of key feature genes in different cell subpopulations identified by PCA. The color gradient from purple to yellow corresponds to low to high expression levels. (**B**) UMAP visualization before and after batch effect correction. Left: Cell distribution before correction, showing a clear difference between batches. Right: Distribution after integration and correction, where the batch effect disappeared, and the cells were well mixed. (**C**) UMAP phase plot of cells by cell cycle gene expression. The cells are colored based on which phase of the cell cycle they have been assigned: G1 (red), S (blue), and G2/M (green). It indicates the position of the cells within the UMAP space. (**D**) UMAP plot of all integrated cells. Each dot represents one cell, and its color indicates the cluster it belongs to, illustrating the differences among the cells and the groups they are part of.

Firstly, we investigated whether the cell cycle stage affected cluster formation by mapping phases (G1, S, G2M) onto the UMAP embedding (Figure 2C). All phases were mixed up inside the UMAP space and did not make separate phase-specific groups, which suggests that how the cells were divided was not the main reason for the big differences between groups of cells. But there was some local enrichment for certain phases in some clusters, which means different levels of cell division happened in each group. PCA applied to the normalized data indicated that the first few principal components accounted for most of the expression variation (Figure 2A), which verified that this low-dimensional representation preserved most of the biologically meaningful information and was appropriate as a foundation for subsequent analyses. UMAP and t-SNE visualizations both showed good integration of cells among different samples in the reduced-dimensional space without clear separation based on sample origin, indicating successful batch integration (Figure 2B,D).

### 2.3. Cell Annotation Results

**Figure 3:**
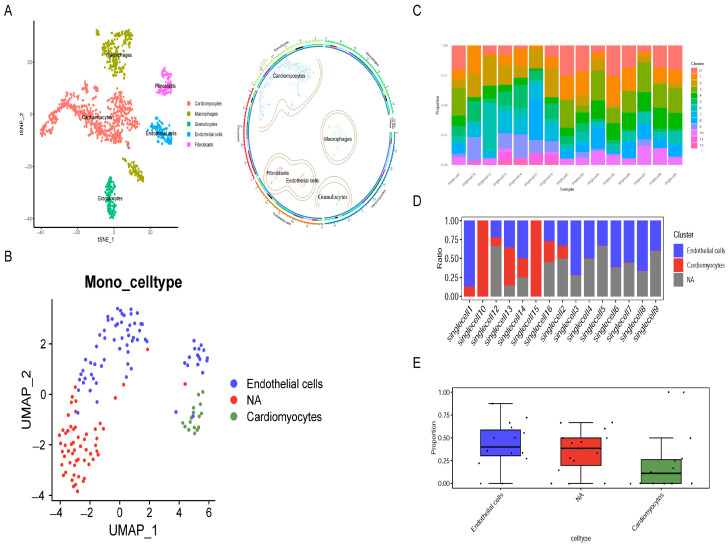
(**A**) Visualization of the result from cell type annotation. Left panel: UMAP plot showing the overall distribution of annotated cell types (colored). Right panel: Circos plot that shows the relationship and proportional composition of different cell types. (**B**) UMAP plot of mononuclear/endothelial-related cell subpopulations. Further division shows endothelial cells (blue), cardiomyocytes (green) and unannotated cells (“NA”, red). (**C**) Stacked bar chart displaying the cellular makeup of every sample. Different colors stand for various groups of cells, so we can tell, at a glance, how the mix of cells varies from one sample to another. (**D**) Heatmap that shows the percentage of each kind of cell that can be found in every sample. The color intensity and fill proportion show the percentage of endothelial cells, cardiomyocytes, and unannotated cells in each sample. (**E**) Boxplots that show how the three different cell types (endothelial cells, unannotated cells, cardiomyocytes) are distributed among all samples, which allows for a comparison of the relative amount of each type of cell.

A systematic comparison of the relative amounts of different cell populations in all samples was performed through a stacked bar graph (Figure 3C), revealing significant differences among samples: each sample has a distinct cellular composition. Take Cluster 0 (pink) as an example, it has the highest percentage in both singlecell5 and singlecell12, whereas Cluster 7 (blue) is the predominant subpopulation in singlecell15. Dynamic changes in the proportions of subpopulations were observed. Some subpopulations, such as Cluster 3 (olive green), were present in several samples, but there was considerable variation in the proportions of these subpopulations between samples, with the lowest proportion occurring in singlecell3 and singlecell7. Rare subpopulations were observed. A few subpopulations, such as Cluster 12 (bright pink), were present only in certain samples at low proportions, suggesting unusual cell types with specific functions or transient states. The global distribution of cell types was visualized by t-SNE and Circos plots (Figure 3A). Based on the annotation results, the cells within the cardiac tissue were divided into five major categories according to their properties: cardiomyocytes (Red), which comprised the largest group, resided on the left side of the t-SNE map and exhibited high consistency; macrophages (yellow–green) formed two distinct clusters on the top and bottom sections, possibly reflecting varying functions; granulocytes (cyan) formed a tight cluster at the bottom; endothelial cells (blue) were located on the right side; and fibroblasts (purple) were found in the upper-right region. There were clear boundaries between the groups, which indicates that the cell annotation was fairly accurate. To examine the heterogeneity in endothelial cells further, another UMAP analysis was performed on the annotated endothelial cells (Figure 3B). The results showed that endothelial cells could be divided into different subpopulations. The blue cluster represents cells with normal endothelial features; some cells are labeled as “NA” (red), which means these cells may be transitional cells or rare subpopulations with specific molecular marks. A small number of cardiomyocytes (green) were also observed in this analysis, which could be due to contamination during cell sorting or temporary state changes, providing valuable clues for studying the functions of different types of endothelial cells. Furthermore, Figure 3D,E show the even distribution of endothelial cells and cardiomyocytes across all clusters and samples.

### 2.4. Co-Expression Analysis

**Figure 4:**
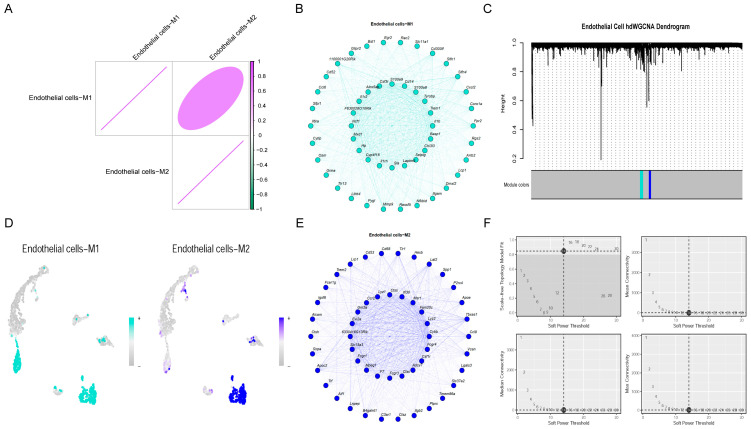
(**A**) Scatter plot showing correlation of gene expression between endothelial M1 and M2 subpopulations. The color bar on the right shows how many points there are at each location; the purple parts show where lots of different genes have similar levels of activity. (**B**) Endothelial M1 subpopulation gene co-expression network. Node (gene) size and edge thickness indicate the degree of connection and co-expression correlation. (**C**) Clustering dendrogram of the endothelial cell hdWGCNA analysis. The bottom of the color bar shows co-expression modules; the blue module is the biggest. (**D**) Distribution plot of the gene expression of endothelial M1 and M2 subpopulations. The color gradient indicates the expression level. (**E**) Gene co-expression network for the endothelial M2 subpopulation, with a different interaction pattern. (**F**) Soft-thresholding analysis of hdWGCNA. Plots for scale-free topology model fit and mean connectivity, which will help us find the best soft-thresholding power.

We used HDWGCNA to make a gene co-expression network for endothelial cells (soft threshold power = 14, Figure 4F), and then identified and focused on two major co-expression modules (Figure 4C)—Endothelial cells-M1 and Endothelial cells-M2 (Figure 4B,E). Genes from both groups had a large amount of module membership (kME). Hub gene screening in the Endothelial cells-M1 module indicated that Il1b was at the top with a kME value of 0.8581, suggesting that it is the most crucial hub gene in this module and may play a prominent regulatory role within the module’s biological process. Trem1 (kME = 0.8337) and Tyrobp (kME = 0.8132) followed, both of which were deemed core hub genes because their kME values exceeded 0.8. Additionally, genes such as S100 calcium binding protein A8 (S100A8) (kME = 0.7957), Cluster of differentiation 14 (Cd14) (kME = 0.7904), S100A9 (kME = 0.7898), and Csf3r (kME = 0.7877) all had high module membership, and together they formed the core gene set of the Endothelial cells-M1 module, ensuring the functionality and specificity of the module. In the M2 co-expression module, Cybb had the highest intramodular connectivity (MM = 0.898), so it was the central hub gene. This module also contained several other highly connected genes, such as Lyz2 (MM = 0.873), Fam20c (MM = 0.840), Msr1 (MM = 0.820), Ifi30 (MM = 0.809), Ctss (MM = 0.801), Lyz1 (MM = 0.778), Ccr2 (MM = 0.778), Gm2a (MM = 0.765), and Evi2a (MM = 0.765). These core genes’ high connectivity shows how important they are in keeping the M2 endothelial cell’s work network together. As a central hub, Cybb may have a substantial effect on the biological functions of this subpopulation through regulating pathways related to oxidative stress and inflammation. The correlation and expression pattern of M1 and M2 modules can be seen in Figure 4A,D.

### 2.5. Characteristic Features of Differentially Expressed Genes in Endothelial Cell Subgroups

The endothelial cell subpopulations showed different expression patterns, and the upregulated genes belonged to several functional categories. From the 102 genes that had increased expression, S100A8 (avg_log_2_FC = 2.6808), Apold1 (avg_log_2_FC = 1.2854), and Nfkbia (avg_log_2_FC = 1.2657) were most highly upregulated. These genes were grouped as follows according to their functions. Endothelial identity and cell–cell junctions: High levels of well-established markers, such as Pecam1, Cdh5, and Robo4, showed that the endothelial cells were pure and had the right identity. Inflammatory and immune signaling: Genes such as S100A8, Cd14, and Nfkbia indicated that active inflammatory pathways and immunomodulatory activity took place inside the endothelial cells. Cytoskeletal structure and metabolic adaptation: Increased Anxa2, Tagln2, and Pkm showed that there was some change in how the cells were organized and how they used energy after the injury.

### 2.6. Endothelial DEGs and HDWGCNA Co-Expression Module Genes

Intersection Analysis: To determine which genes are the most important for controlling the function of endothelial cells, we examined where the genes that changed a lot in endothelial cells overlapped with groups of genes working together using the HDWGCNA method. We found 54 such genes in total. These genes have both “important difference” and “working together to control other genes” characteristics. They make a range of molecules that assist in endothelial cell functions. Looking at how these overlapping genes were expressed over time revealed a clear pattern: 40 of them (74.1%) were much higher than normal, while just 14 (25.9%) were lower, showing that the main regulation of major endothelial functions relies on these increased genes working together. Among the 40 upregulated intersecting genes, five genes had an avg_log2FC > 2.5 and were considered specific and highly expressed core genes in endothelial cells: S100A8, Ctss, Spp1, Apoe, and Lyz1. S100A8 is a marker of inflammation that helps recruit and activate immune cells by binding to receptors. Ctss and Lyz1 participate in lysosomal work and cutting proteins, which are related to altering the outside parts of cells and sending messages about getting sick. Spp1 manages how cells connect, move, and transform into bone-building cells, aiding in maintaining the small blood pathway. Apoe is involved in lipid metabolism and cholesterol transportation; its abnormal expression is closely linked to vascular endothelial injury and inflammation development.

### 2.7. Enrichment Analysis

**Figure 5:**
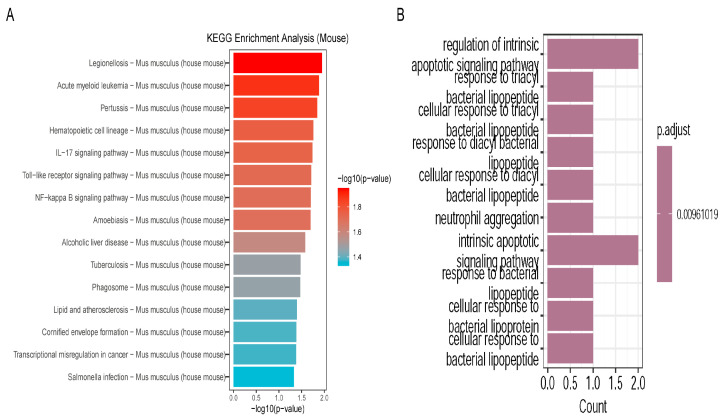
(**A**) Bar graph of KEGG pathway enrichment analysis of differentially expressed genes. It displays the most significantly enriched pathways, where the x-axis indicates −log_10_(*p*-value). (**B**) Bar graph of GO biological process (BP) enrichment analysis for differentially expressed genes. The X-axis represents the number of enriched genes, and the most important terms are on the right side.

GO enrichment analysis (Figure 5B) showed that the core endothelial genes were mainly related to biological processes such as inflammatory response, immune cell activation, and regulation of apoptosis. Regulation of the intrinsic apoptotic signaling pathway is the most significant one. Inflammatory-related processes, such as “response to bacterial lipopeptide”, “neutrophil aggregation”, etc., were also found. These results suggest that endothelial cells start innate immune reactions and change cell death to keep blood vessels stable after injury. Kyoto Encyclopedia of Genes and Genomes (KEGG) pathway analysis (Figure 5A) further revealed the signaling networks that contain these core genes. The IL-17 signaling pathway was identified as the most enriched pathway. This pathway acts as an important hub of the inflammatory cascade. It activates the downstream Nuclear Factor-kappa B (NF-κB) signaling pathway, which then promotes the expression of pro-inflammatory cytokines and chemokines, recruits and activates neutrophils, and intensifies the local inflammatory response. Co-enrichment of the Toll-Like Receptor (TLR) and NF-κB signaling pathways implies that endothelial cells use them as a linked signaling network to trigger inflammation.

### 2.8. Core Gene Screening and AI-Based Protein Interaction Analysis

**Figure 6:**
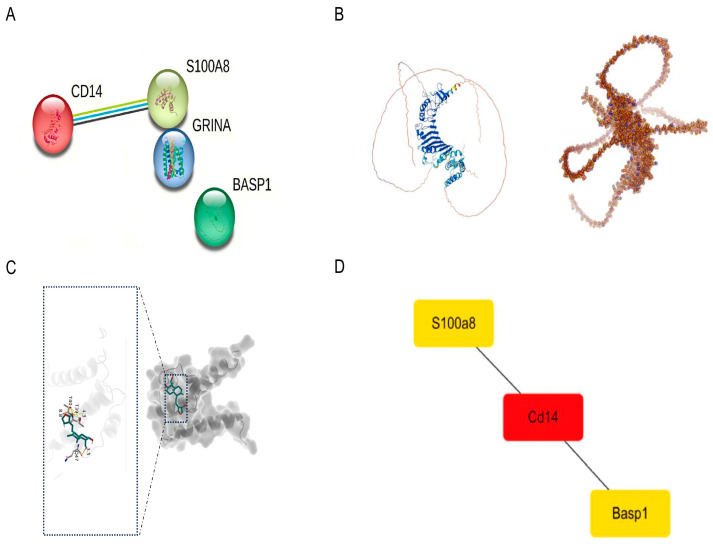
(**A**) Protein–protein interaction network visualization of core genes. Nodes (colored and shaped) are genes; edges are interactions. (**B**) Some important protein 3D structure models. Left: CD14 ribbon diagram. Right: Surface presentation of S100A8, showing its spatial configuration. (**C**) Small molecule–target protein molecular docking result. Overall view and close-up view of the binding pose and key interaction sites. (**D**) S100A8, CD14, and BASP1 interact directly with one another in a network diagram. CD14 is highlighted as a central node, indicating that it may have a collaborative role in this complex system.

To break down the main process of endothelial inflammatory regulation on a molecular scale, we combined PPI network analysis and AI-aided 3D structure prediction to systematically confirm the functional relationship and physical connection of important genes. Constructing a PPI network via the STRING database (Figure 6A), followed by carrying out topological analysis of the network using the cytoHubba plugin within Cytoscape, revealed that CD14 was a central hub protein (red node) within the network, which was directly linked to upstream S100A8 and downstream BASP1, creating a clear signal axis (Figure 6D). CD14 binds to S100A8, so this interaction network is one of the major pathways from inflammation to cell fate choices. And it shows where these two molecules work together at the PPI level to control endothelial inflammation. We employed AlphaFold3 to predict the complex structure of S100A8 and CD14 and investigate whether there is a potential interaction between these two proteins (Figure 6B). The predicted model has a global confidence score (pTM) of 0.5 and an interface confidence score (ipTM) of 0.15. According to AlphaFold’s criteria, these scores indicate a moderate-to-low confidence level, suggesting that the predicted overall fold and interface point to a possible binding region. Structural visualization shows that the predominantly β-sheet region of S100A8 may form contacts with the extracellular domain of CD14. The predicted binding interface involves hydrophobic and charged amino acid residues, potentially stabilized by hydrogen bonds and hydrophobic forces. This computational prediction provides preliminary structural clues for the potential interaction between S100A8 and CD14, laying the foundation for further experimental validation. In summary, PPI network analysis and AI-driven protein structure prediction together suggest that S100A8 may be a core regulatory node in the endothelial microenvironment during MI/R injury.

### 2.9. Transcriptional Changes After Virtual S100A8 Knockout

**Figure 7:**
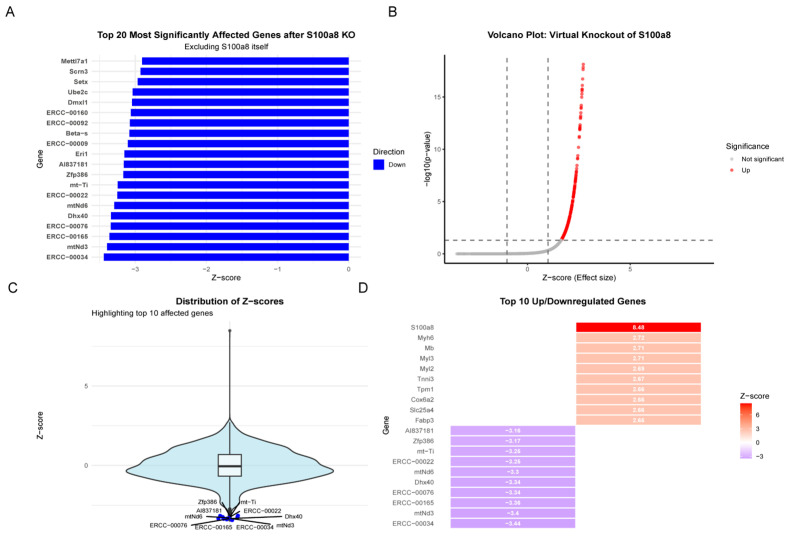
(**A**) Bar chart of the top 20 most affected genes after a virtual S100A8 knockout, excluding S100A8 itself. Z-scores show how much the expression has changed. (**B**) Volcano plot for differentially expressed genes following virtual S100A8 knockout. X axis: effect size; Y axis: −log10(*p*-value). Red dots: significantly upregulated genes; gray dots: non-significant genes. (**C**) Violin plot of X-score distribution for all genes that were virtually knocked out of S100A8. Bottom panel: Top 10 most affected genes. (**D**) Heatmap of the top 10 most important gene expression changes (Z-scores) after virtual S100A8 knockout. Colors go from red (very positive Z-score) to purple (very negative Z-score).

To study the role that S100A8 plays inside the endothelial microenvironment during MI/R damage, we simulated its loss of function by using a “virtual knockout” method. Then, we observed how the whole set of genes (what we call the genome) changed because this S100A8 virtual knockout brought about considerable changes in two major gene clusters (Figure 7A). The first cluster consisted of genes related to cardiac muscle contraction and energy metabolism, such as Myh6, Myh7, Tpm1, Cox4i2, and Atp2a2 (Figure 7D). Changes in these genes should directly impact the heart’s ability to contract and produce energy. The second cluster had some genes that were connected with mitochondria and RNA processing, such as mt-Tf, mt-Tl1, and Dhx40, and also some ERCC quality-control genes, which are part of the process of mitochondrial translation and RNA splicing/modification—important processes that maintain cell metabolism and accurate DNA. These results place S100A8 at the center of the regulation of MI/R injury. These findings both verify that it plays a pivotal role in the endothelial microenvironment and give direct proof of its function. Target selection for virtual knockout and the resulting expression change is shown in Figure 7B,C.

### 2.10. Small Molecule Drug Screening and Molecular Docking Validation

Andrographolide (AG) was identified as the best choice from our screening. It is a natural compound that comes from Andrographis paniculata. Based on the literature, it has anti-inflammatory, antioxidant, and anti-apoptotic properties. As per the KEGG pathway analysis, andrographolide seems to affect inflammatory signaling, including the IL-17 pathway, which might improve the inflammatory microenvironment during MI/R injury. Following this logic, we chose andrographolide as a possible intervention aimed at S100A8 and carried out molecular docking to assess how it binds. The docking results (Figure 6C) showed that there was a binding energy of −5.9 kcal/mol between andrographolide and S100A8, which indicated spontaneous and stable complex formation. Binding mode analysis shows that andrographolide interacts with S100A8 via multiple non-covalent interactions. The main polar contact is a hydrogen bond with the side-chain amide of Gln47 (distance ~3.5 Å) and another hydrogen bond with the backbone carbonyl of Thr39.

### 2.11. Andrographolide Inhibits the S100A8/IL-17 Axis and Reduces Hypoxia/Reoxygenation (H/R) Injury on Cardiac Microvascular Endothelial Cells

**Figure 8:**
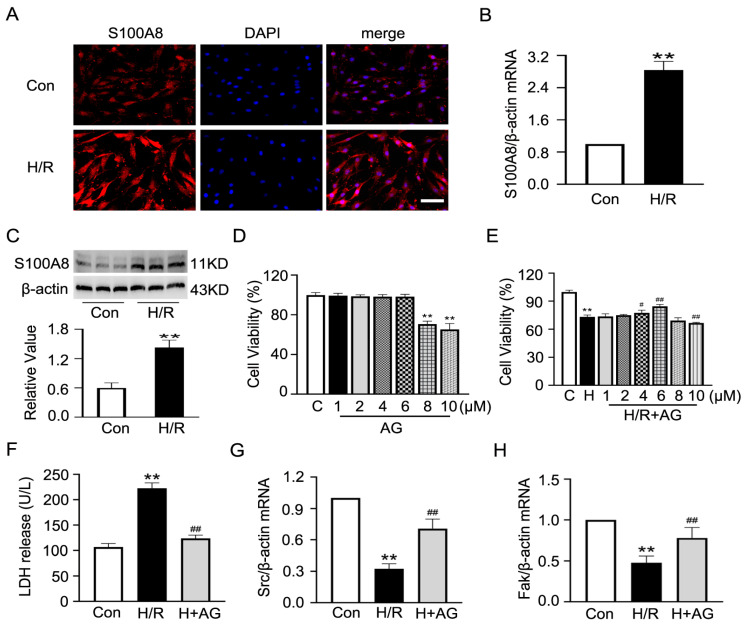
Andrographolide reduces H/R-induced CMEC injury. (**A**–**C**) Immunofluorescence staining, mRNA and protein expression of S100A8 (*n* = 3). Scale bar: 100 µm. (**D**,**E**) Cell viability was detected by the CCK-8 method under normoxia and H/R conditions after AG treatment with different concentrations (1, 2, 4, 6, 8, 10 μM). (**F**) LDH release measurement. (**G**,**H**) qRT-PCR for mRNA expression of Src and Fak. Data are presented as mean ± SD (*n* = 6). “Con” stands for the control group, “H/R” or “H” indicates the H/R group, and “H + AG” means the H/R group treated with AG. ** *p* < 0.01 compared to the Con group; ^#^ *p* < 0.05 and ^##^ *p* < 0.01 compared to the H/R or H group.

**Figure 9:**
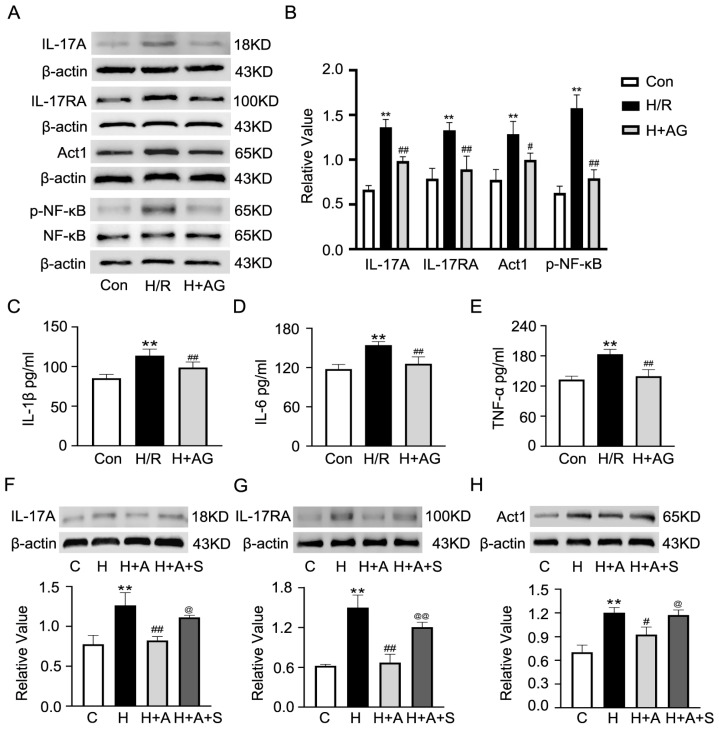
Andrographolide inhibits the S100A8 downstream IL-17 signaling pathway and reduces the inflammatory response of H/R-induced CMECs. (**A**,**B**) The expressions of IL-17A, IL-17RA, Act1, and NF-κB proteins were determined by Western blot (*n* = 3). (**C**–**E**) The levels of IL-1β, IL-6, and TNF-α were determined through ELISA. (**F**–**H**) The expressions of IL-17A, IL-17RA and Act1 proteins were determined by Western blot in co-transfection with the overexpression S100A8 plasmid (*n* = 3). Data are presented as mean ± SD (*n* = 6). “Con or C” stands for the control group, “H/R” or “H” indicates the H/R group, and “H + AG or H + A” means the H/R group treated with AG. “H + A + S” means the H/R group treated with AG and overexpression of S100A8. ** *p* < 0.01 compared to the Con group; ^#^ *p* < 0.05, ^##^ *p* < 0.01 compared to the H/R or H group, ^@^ *p* < 0.05, ^@@^ *p* < 0.01 compared to the H + A + S group.

Our single-cell sequencing data indicated that S100A8 was the highly expressed marker gene in endothelial cells under I/R conditions. And it was also verified through cultured CMECs that H/R led to a notable rise in S100A8 levels detected via fluorescence, protein, and mRNA (Figure 8A–C). Since andrographolide (AG) is known to have anti-inflammatory effects, we aimed to determine if it could also help with H/R-induced inflammation. Through the CCK-8 test, first, we found that 4 and 6 μM AG under normoxia conditions did not affect the normal growth of CMECs, but 8 and 10 μM AG were toxic (Figure 8D). After H/R stress, 4 and 6 μM AG increased cell survival, whereas 10 μM AG decreased cell survival (Figure 8E). So, we chose 6 μM AG for all further experiments. At this dose, AG greatly reduced LDH release (Figure 8F) and recovered the expression of barrier-related genes *Src* and *Fak*, which had been inhibited by H/R (Figure 8G,H). As per the earlier KEGG analysis linking these genes with the IL-17, TLR and NF-κB pathways (Figure 5A), we checked how AG affected the IL-17 cascade. Western blot showed that H/R raised the amounts of Interleukin 17A (IL-17A), its receptor Interleukin-17 receptor A (IL-17RA), the adapter NF-κB activator 1 (Act1), and phospho-NF-κB, all of which were decreased by AG co-treatment (Figure 9A,B). AG also blocked the rise in pro-inflammatory cytokines IL-1β, IL-6, and TNF-α due to H/R (Figure 9C–E). Furthermore, to further demonstrate that AG inhibited the IL-17 signaling pathway through the S100A8 molecule, we overexpressed the S100A8 plasmid in CMECs while simultaneously administering AG. As shown in Figure 9F–H, the AG-induced reduction in the expression of IL-17A, IL-17RA and Act1 under H/R conditions was partially restored upon co-transfection with the overexpression S100A8 plasmid. Taken together, these results suggest that AG protects CMECs against H/R-induced injury through inhibition of the S100A8-mediated IL-17 pathway and decreased inflammation.

### 2.12. AG Reduces Inflammation and Maintains Microvascular Integrity in the Mouse I/R Model

**Figure 10:**
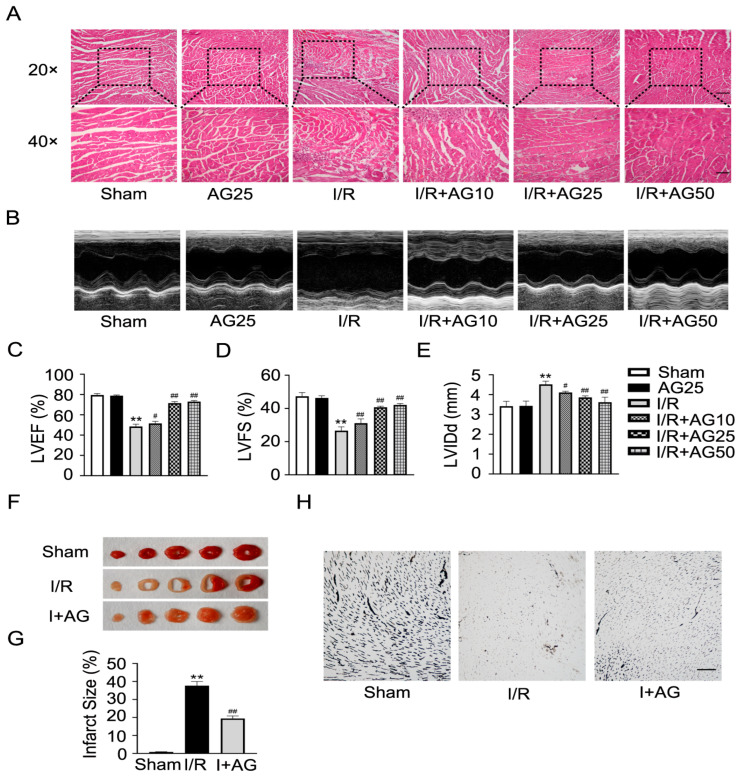
AG can alleviate cardiac microvascular I/R injury. (**A**) H&E staining of mouse heart tissue to observe pathological changes. Inset: 20× and 40×. Scale bars: 100 µm and 50 µm. (**B**–**E**) Representative echocardiograms and LVEF, LVFS, and LVIDd values for the Sham, I/R, and I/R + AG groups. (**F**,**G**) TTC staining was used for infarct size assessment and quantification in I/R model mice (*n* = 3). (**H**) Gelatin–ink staining for microvascular perfusion. Scale bar = 100 µm. Data are presented as mean ± SD (*n* = 6). “Sham” refers to the sham-operated group, “I/R” refers to the I/R injury group, and “I + AG” refers to the I/R group treated with AG. ** *p* < 0.01 compared to the Sham group; ^#^ *p* < 0.05, ^##^ *p* < 0.01 compared to the I/R group.

**Figure 11:**
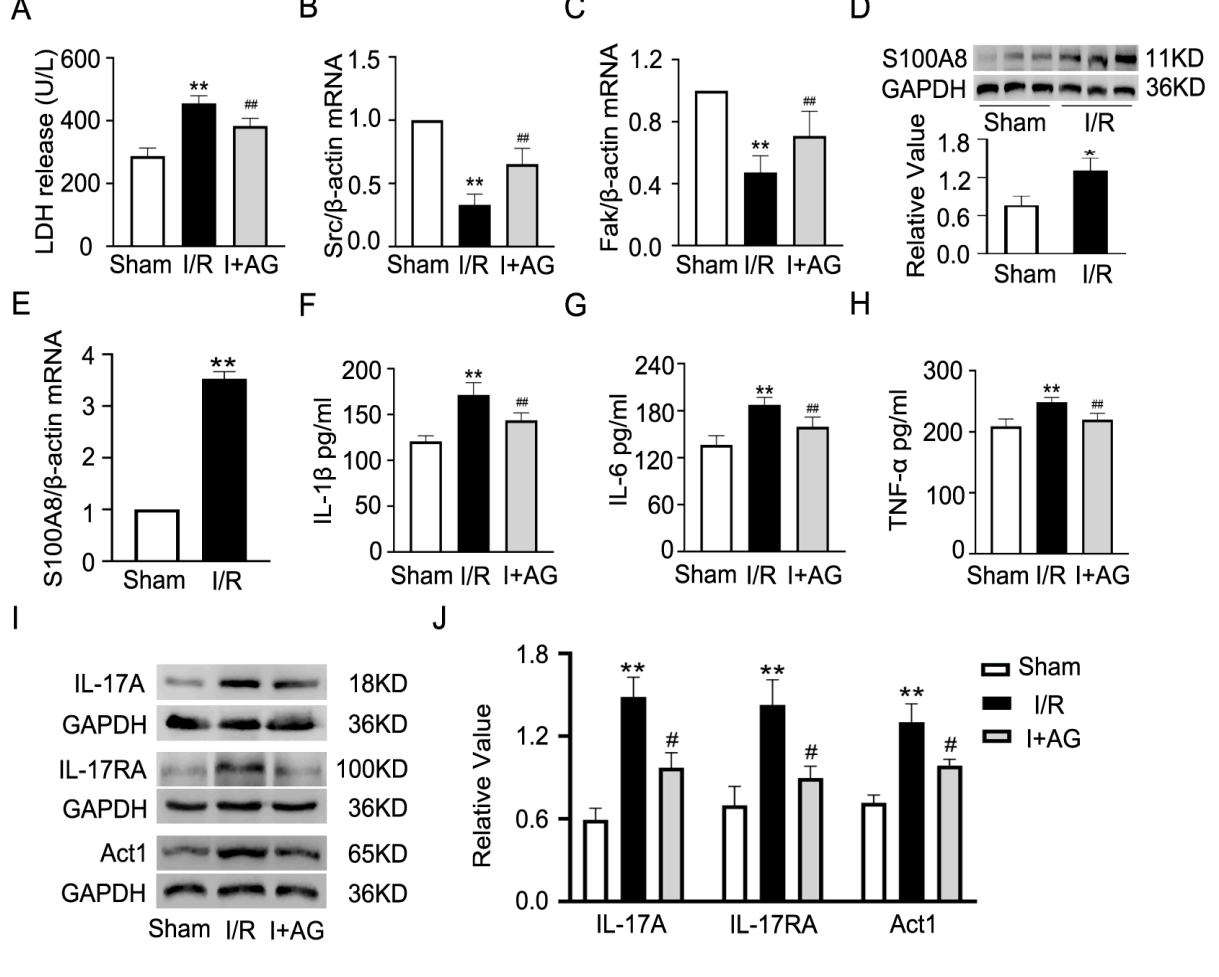
AG can inhibit inflammation and the IL-17 pathway caused by cardiac microvascular I/R injury. (**A**) Measurement of LDH release. (**B**,**C**) mRNA expression of Src and Fak detected by qRT-PCR. (**D**,**E**) S100A8 mRNA and protein detection (*n* = 3). (**F**–**H**) IL-1β, IL-6, and TNF-α concentrations measured by ELISA. (**I**,**J**) Protein expression of IL-17A, IL-17RA, and Act1 detected by Western blot (*n* = 3). Data are presented as mean ± SD (*n* = 6). “Sham” refers to the sham-operated group, “I/R” refers to the I/R injury group, and “I + AG” refers to the I/R group treated with AG. * *p* < 0.05 and ** *p* < 0.01 compared to the Sham group; ^#^ *p* < 0.05 and ^##^ *p* < 0.01 compared to the I/R group.

Then, we checked whether AG could prevent cardiac microvascular I/R injury in vivo. The mice were administered AG (10, 25, or 50 mg/kg/day) for 7 days before undergoing I/R surgery, during I/R surgery and the day after surgery. After 24 h of reperfusion, the hearts were stained with H&E, and it was found that AG treatment in a dose-dependent manner decreased red blood cell aggregation, thrombus formation, inflammatory cell infiltration, and myofiber disarray (Figure 10A). Echocardiography showed improved function: AG-treated mice had improved LVEF and LVFS and a shortened LVIDd compared to the I/R group (Figure 10B–E). Since 25 mg/kg was good (*p* < 0.01) and did not have any toxic effects, this dose was used for all the other experiments. The infarct size was noticeably smaller in the 25 mg/kg AG group under I/R based on 2,3,5-Triphenyltetrazolium chloride (TTC) staining (Figure 10F,G). The gelatin–ink perfusion imaging showed that the hearts treated with AG had better maintained microcirculatory blood flow in I/R injury (Figure 10H). AG also reduced the I/R-induced LDH release (Figure 11A) and recovered cardiac mRNA levels of Src and Fak (Figure 11B,C). AG administration also blocked the I/R-induced rise in S100A8 mRNA and protein in heart tissue (Figure 11D,E). ELISA tests showed that AG greatly reduced the levels of IL-1β, IL-6, and TNF-α in the myocardium of I/R-damaged mice (Figure 11F–H). Finally, AG administration inhibited the expression of IL-17 pathway components such as IL-17RA, IL-17A, and Act1 (Figure 11I,J). In a nutshell, AG therapy can reduce cardiac inflammation, restrict microvascular dysfunction, and improve the recovery function after I/R injury through the S100A8/IL-17 axis.

## 3. Discussion

MI/R injury is a complex pathophysiological process, where different types of cells and signals interact with each other in complex ways over space and time, resulting in damage and dysfunction [12]. Scientists have researched cardiomyocytes for many years; they have studied how these cells die, what happens when their energy supply ends, issues with calcium dysregulation, and when the heart stops beating correctly [13]. While many cardioprotective strategies show promise from these studies, there remains what is known as a “translational gap”—methods that work in animals do not necessarily translate to humans. And then this difference creates a fundamental question: Did we miss something more important that happened before the injury began?

From a clinical perspective, the “no-reflow” phenomenon indicates that successful reperfusion means restoring flow at the microvascular level. The rapid morphological and behavioral changes in endothelial cells—the critical interface between blood and tissue—position them as potential primary instigators of reperfusion injury. In this study, we shifted focus from cardiomyocytes to endothelial cells with single-cell transcriptomics, AI-driven simulations, and various experiments [14]. We found that S100A8 was a significant molecular hub that became activated shortly after MI/R occurred, and we identified a novel type of positive feedback loop for inflammation via the IL-17 signaling pathway [15]. Additionally, andrographolide (AG) was screened and validated as a natural compound intervention targeting this axis. The four major points of this section are a paradigm shift and mechanism insight, methodological integration value, translational implication, and study limitations.

Paradigm shift: Endothelial cells are early drivers, not passive bystanders. Traditionally, endothelial dysfunction was considered to be caused by cardiomyocyte damage. Based on our single-cell data, we see fast and specific changes in how genes work in cardiac endothelial cells within 1–3 days of reperfusion, so these covering cells become the center of the first bad reaction. And this change moves the endothelium from being simply a part of the inflamed area into something that raises alarms all on its own. We found that the most important controlling group was near S100A8 by doing a co-expression network analysis. S100A8 is a well-known DAMP protein that was thought to originate primarily from infiltrating leukocytes. Here, we show that endothelial cells themselves are a major early source of S100A8, changing the way we think about them in three ways: endothelial cells act as sentinels, turning ischemia/reperfusion stress into a biological alarm and potentially setting off the inflammatory cascade [16].

The novelty of this work lies in revealing the significance of S100A8 within endothelial cells—a discovery that expands its functional role from a conventional inflammatory effector to an initiating trigger of endothelial injury, extending far beyond its well-recognized role in inflammation. This paradigm shift fundamentally alters our understanding of how MI/R injury begins and, consequently, how it might be more effectively intercepted.

Local origin of inflammation: Microvascular inflammation starts within the vessel walls, indicating that endothelial cells are the primary initiators of the first inflammatory response. S100A8 has two roles. When it is secreted, S100A8 functions as an alarmin for intercellular communication. But if it builds up inside the cell, it triggers a pro-inflammatory switch in endothelial cells, which means that S100A8 also has an important regulatory function inside the cell. Protein interaction network analysis and AlphaFold3-based structural prediction of the S100A8-CD14 complex show that S100A8 is an early injury signaling node. The mechanism is a self-amplifying inflammatory loop within endothelial cells [17].

Our second major finding is that S100A8 amplifies its own signaling through the IL-17 pathway in endothelial cells. KEGG pathway enrichment analysis and experimental results indicate that S100A8 signaling upregulates the expression of IL-17 receptors, suggesting that endothelial cells may exhibit enhanced sensitivity upon subsequent IL-17 stimulation. Furthermore, activated endothelial cells themselves can produce IL-17, suggesting a potential positive feedback regulatory loop: S100A8 → Enhanced IL-17 Pathway → Increased IL-17 Sensitivity → Stronger Inflammatory Response → More S100A8/IL-17 Production. This cell-autonomous amplification circuit provides a new mechanistic perspective for understanding the rapid initiation and persistence of vascular dysfunction following MI/R injury, as well as the sharp upregulation of pro-inflammatory cytokines (TNF-α, IL-1β, IL-6) [18,19] and adhesion molecules. Virtual knockout of S100A8 disrupted this loop and significantly altered the expression of multiple genes related to myocardial contraction and energy metabolism, further supporting the key role of S100A8 in the early initiation of microvascular injury.

Therapeutic translation: Andrographolide as an exact microvascular protector. We placed andrographolide (AG) [20], a traditional anti-inflammatory agent, at the position of a “microvascular protector” targeting the initial endothelial inflammatory focus. In animals, AG treatment improved cardiac function [21], reduced myocardial infarct size, and, most importantly, preserved microvascular integrity, as evidenced by gelatin–ink staining. Mechanistically, AG dose-dependently inhibited S100A8 upregulation and blocked the downstream over-activation of the IL-17 pathway in endothelial cells [22], indicating that AG specifically targets the S100A8/IL-17 axis [23] rather than having nonspecific anti-inflammatory effects [24]. Although we do not know how AG is metabolized [25,26], what its specific molecular targets are, or the best time for administration, this study provides a basis for us to consider using AG to treat microcirculatory disorders following MI/R.

Limitations and Future Directions: (1) Spatiotemporal cell–cell communication: In the future, we will have to use spatial transcriptomics to identify how S100A8-driven endothelial signals alter macrophage polarization, neutrophil recruitment, and fibroblast activation at the site of inflammation. AG’s exact target engagement: AG binds S100A8 according to molecular docking, but SPR or ITC biophysical validation is needed. More research needs to be done to determine if AG changes how much S100A8 is made, changed into proteins, sent out of cells, or paired up with another S100A8 molecule. (2) Clinical correlation and translation: Levels of S100A8, IL-17-related cytokines, and endothelial biomarkers should be measured in AMI patients and correlated with microvascular obstruction and clinical outcomes. Large-animal studies (e.g., porcine MI/R models) have to be done to figure out the best treatment time (before, during, or after reperfusion) and whether it works well when combined with other treatments (such as drugs that prevent blood clots from forming). (3) Proof-of-concept nature of therapeutic potential: Although molecular docking suggests that AG can bind to S100A8, its direct interaction needs to be validated by biophysical techniques such as surface plasmon resonance or isothermal titration calorimetry. Furthermore, the mechanisms by which AG affects S100A8 synthesis, post-translational modification, secretion, or dimerization remain unclear. The current study positions AG as a candidate targeting molecule that remains at the proof-of-concept stage. Future efforts should systematically evaluate its pharmacokinetic profile, endothelial cell targeting specificity, optimal dosing regimen, potential off-target effects, and long-term safety to advance its translation from basic research to preclinical development.

In short, by focusing on endothelial cells and using single-cell genomics combined with AI simulations and experimental verification, we found that S100A8 was an early hub in MI/R injury, identified a self-amplifying inflammatory loop driven by IL-17, and proposed andrographolide as a potential therapeutic target. This integrated approach not only provides new mechanistic insights into the pathogenesis of MI/R injury but also establishes a foundation for developing targeted therapies to preserve microvascular function and improve clinical outcomes in ischemic heart disease.

## 4. Materials and Methods

### 4.1. Data

In order to give a full account of the cell–cell communication networks and molecular driving events during the acute repair phase following cardiac ischemic injury, this study used the public single-cell transcriptomic dataset GSE146285. This dataset is from a mouse MI/R model that gathers heart tissues at different times after the heart stops receiving blood. To know which part of the body reacts first to pain and which part reacts first to becoming red and swollen, this study picked out two specific days: the first day after the pain happened (Day 1 PI) and the third day after (Day 3 PI). These are the moments when there is very quick swelling and when things begin to improve; therefore, they are key times when cells alter their form, many helper cells become involved, and some initial indicators of hardening show up—this aids our comprehension of why the body responds to being injured. For each sample, we obtained raw sequencing data through the 10× Genomics Chromium platform and created gene expression matrices as usual. All bioinformatics analysis and AI computing related to this study were carried out on a particular workstation equipped with a powerful GPU. It has an Intel Core i7-650H series CPU, 64 GB DDR4 RAM, and enough memory bandwidth and multi-tasking parallel processing capabilities to deal with big single-cell transcriptomic data. Complex deep learning models for proteins and molecular docking have become much faster thanks to the NVIDIA GeForce RTX 4050 graphics card; thus, the outcomes of computations remain constant and can be replicated.

### 4.2. Single Cell Data Integration and Quality Control Analysis

Data processing: Each sample was treated as an individual analysis object, and the same quality control standards were applied to each one [27]: only genes present in at least three cells were retained, and each cell needed to express at least 200 genes. This removed the bad cells and the background noise. Data reading and conversion parameters were the same for all samples. After the first round of quality control, we adopted a systematic integration approach to incorporate all sample data into a single analytical entity. By integrating so that each cell’s original ID has the sample origin info at the front, we could follow cell lineages correctly and avoid any ID clashes between different samples. Finally, the combined dataset was saved in a normal format to have one data structure for all the samples, cells, and gene expressions. Quality control step: The Seurat [28] package (version 4.4.0) was used for systematic pre-processing and quality control of the single-cell RNA sequencing data. First, Seurat functions were used to ensure that all gene names were distinct and separate so that each gene could be recognized individually. Then, we picked out the nice cells based on some important numbers from Seurat, such as the amount of hemoglobin gene expression, to check if any red blood cells had been mixed in. A stricter filter threshold was applied, keeping cells with nCount_RNA ≥ 1000, nFeature_RNA between 200 and 5000, and Hb gene expression ≤ 3%. Expression data was normalized using Seurat’s NormalizeData function (log normalization), and the top 2000 most variable genes were found with FindVariableFeatures. Then, we did PCA with Seurat, picked the top 30 PCs based on how much they added up when we added them together, and used UMAP and t-SNE to make the data easier to see without making it linear. The Harmony algorithm was used to remove technical batch effects among different samples through the RunHarmony function of the Seurat package (v4.4.0). The first 30 principal components were chosen as input, and the sample origin was used as the grouping variable for adjustment.

### 4.3. Single-Cell Atlas Annotation and GPTCelltype Subpopulation Analysis

The Seurat package was applied initially for quality control, normalization, and feature selection on the raw data. To remove the batch effect, we used the Harmony algorithm to integrate and correct the data among different samples. Then, PCA was carried out, and the best number of principal components (PC = 15) was determined using an elbow plot. A k-nearest neighbor graph was created with these parts. Cell subpopulations were identified by using a multi-resolution clustering approach (resolutions from 0.01 to 3.0), and it turned out that the optimal resolution was 0.05 based on the cluster tree. Cell type annotation was done by the SingleR algorithm, which automatically labels cells according to how similar they are to known human/mouse cell types from the celldex package using Pearson correlation. Visualization was done by t-SNE and UMAP dimensionality reduction projection. The GPTCelltype model was used as an automatic labeling tool for cell subpopulations. For endothelial cell clusters, the top 50 uniquely expressed genes for each cluster, as determined by Seurat’s FindAllMarkers function, were selected as the feature gene sets. Then, these gene lists were merged with a cell type background knowledge database to form structured search requests so that the genes could have sufficient expression specificity to be properly labeled [29].

### 4.4. Single-Cell HDWGCNA Analysis

hdWGCNA [30] was applied to create gene co-expression networks from the single-cell transcriptomic data. Endothelial cells were first separated from the annotated single-cell dataset; then, metacells were created to reduce the sparsity of the data. Normalization done and batch effect adjusted with Harmony. A weighted gene co-expression network was constructed with a soft-thresholding power of 14. Co-expression modules were discovered by calculating the topological overlap matrix and performing hierarchical clustering. And lastly, we calculate the module eigengenes (first principal component of each module) and the gene module connectivity (kME values).

### 4.5. Single-Cell Differential Expression and Gene Overlap Analysis

To determine which genes have differential expression across all the cell subpopulations, we used the FindAllMarkers function in Seurat with min.pct = 0.25 and logfc.threshold = 0.25. DEGs were obtained from every subpopulation, duplicates were deleted, and group expression variations were analyzed [31]. Genes that met both the significance filter (adjusted *p*-value < 0.05) and the expression change filter (|log_2_FC| > 0.25) were retained for further analysis. Then, we did an intersection analysis on this filtered DEG group with the hub genes identified by HDWGCNA. After making such a comparison, the genes that matched were considered as good options for further study to find out what they do.

### 4.6. Single-Cell Enrichment Analysis

Gene identifiers were first converted by the mouse gene database (.db). GO enrichment [32] analysis was then carried out using the clusterProfiler package, which included the three main categories of biological process, cellular component and molecular function. From the results, signaling pathway-related words were picked out, and the top 10 most important ones were shown. For KEGG [33] pathway analysis, the WebGestaltR package was first applied, but because there was sometimes instability with the online tool, local KEGG data were then used for internal enrichment analysis. Lastly, the top 15 most enriched pathways were shown as bar plots.

### 4.7. Screening for Core Genes Using AI-Supported Structure Analysis and Validation

We made a mouse protein–protein connection map using STRING (https://cn.string-db.org/ (accessed on 10 October 2025)). We set the confidence level at 0.4 and took out unconnected parts so we could look at important connections. Then, it was shown as a picture and visualized with Cytoscape (version 3.10.1) [34], a computer program that helps us see how things are connected. We used special tools from a part of Cytoscape called cytoHubba to find the most important genes (we call them “hub genes”) based on their positions in the picture. For structural modeling, the full amino acid sequence of the protein of interest was acquired from UniProt. Its 3D conformation was predicted by AlphaFold3 [35], which gets evolutionary constraints from sequence alignments and templates and then refines these constraints via the Evoformer and structure modules to create candidate models. The model with the highest confidence (pLDDT and pTM scores) was chosen as the final structure and displayed using PyMOL (version 3.1.3.1) [36].

### 4.8. Virtual Cell Knockout Model and Molecular Docking

To simulate gene loss of function, we used scTenifoldKnk [37], an unsupervised machine learning tool. Based on wild-type scRNA-seq data, it first constructs a gene regulatory network (scGRN) via tensor decomposition—a technique that factorizes a three-dimensional tensor built from cell-level expression profiles to capture higher-order gene relationships and reduce noise. To mimic the loss of regulatory output of a target gene, it generates a pseudo-knockout network by setting all outgoing edges from that gene to zero while preserving the rest of the network. By comparing the wild-type and pseudo-knockout networks through manifold alignment—which projects both networks into a common low-dimensional latent space and quantifies the displacement of each gene—we calculated the regulatory perturbation for each gene as the magnitude of its displacement. Based on the distribution of these perturbations across all genes, we derived Z-scores and corresponding *p*-values. Genes with |Z| > 1 and *p* < 0.05 were identified as significantly differentially regulated, revealing the functional role of the candidate gene by indicating which genes’ regulatory states are most affected by its loss.

Compound Library and Docking: We made a small-molecule library that has the main gene of the inflammation control path as its center [38]. Candidate compounds were found by searching PubMed and other literature with terms such as “cardiac endothelial cells”, “inflammatory regulation”, “myocardial ischemia/reperfusion injury” and “small molecule drugs”. According to the mechanism-based evidence from the literature, we selected those molecules that have been reported to possess anti-inflammatory and cardioprotective properties. Molecular docking was carried out by AutoDock Vina (version 1.5.7) [39]. The 3D structure of the target protein that had been predicted using an AI-based method was prepared by removing water molecules, adding hydrogen atoms, and optimizing protonation states with the aid of molecular visualization software. Atomic charges were provided, and spatial affinity grids were created via a computer procedure. Ligand structures were reduced for energy and then changed into a format suitable for the docking workflow prior to screening.

### 4.9. In Vitro and In Vivo Validation Experiment of the Endothelial Core Regulatory Hub

#### 4.9.1. Experimental Animals

Adult male C57BL/6J mice (body weight 22–32 g) were obtained from the Laboratory Animal Center of Heilongjiang Bayi Agricultural University. The research followed the Guide for the Care and Use of Laboratory Animals (AAALAC) and received approval from the Experimental Animal Ethics Committee of Heilongjiang Bayi Agricultural University (Approval No. DWKJXY2026016). All mice used in this study were non-genetically modified. The mice were housed under normal laboratory conditions: temperature at 22 ± 2 °C, humidity between 40 and 60%, light/dark cycle of 12 h/12 h, free access to standard rodent chow and water ad libitum [40].

After a 7-day acclimatization period, the mice were randomly assigned to different groups using a computer-generated random number table. Any mice that died during the surgical procedure or prior to the experimental endpoint, as well as those in the I/R group that subsequently failed to show confirmed I/R injury upon histological examination, were excluded from the study. The final sample size (*n* = 6/group) for each group was consistently used for Hematoxylin and eosin (HE) staining, echocardiography, molecular biology and biochemical detection assays. The final sample size (*n* = 3/group) for TTC staining and Western blot was consistent for the Sham, I/R and I/R + AG groups. To minimize potential confounding factors, surgeries for each group were all performed in a randomized sequence, whereas other potential confounders, such as the cage position within the animal room, were not systematically controlled. Blindness was implemented for the outcome assessment. The researchers conducting the surgeries and administering the drugs were aware of the group assignments, but the personnel conducting the subsequent outcome evaluations (echocardiography technicians, tissue pathologists, etc.) were unaware of the group information.

#### 4.9.2. Establishment of Mouse Myocardial I/R Model and Grouping

A myocardial I/R model was made using the LAD ligation method, as described in our previous study [41]. To summarize, the mice were anesthetized by intraperitoneal injection of sodium pentobarbital (30 mg/kg); then, the trachea was intubated and connected to a small-animal ventilator (BL-420I, Taimeng, Chengdu, China). An electrocardiogram was observed throughout the entire process. Left thoracotomy was performed to expose the heart. LAD was ligated proximally with an 8-0 silk suture (Ningbo, China) using a slipknot to cause myocardial ischemia for 40 min. Reperfusion was started by releasing the slipknot and maintained for 24 h. First, the experiment was randomly divided into 6 groups, including Sham, Sham + AG (25 mg/kg), I/R, I/R + AG (10 mg/kg), I/R + AG (25 mg/kg), and I/R + AG (50 mg/kg), and subjected to HE staining and echocardiography assays. Sham group: Suturing was performed under LAD without ligation. All other procedures were the same as those of the model group, and the mice were given the same quantity of saline. I/R group: Only LAD ligation and reperfusion were performed, and the mice were given the same quantity of saline. AG groups: The mice were injected intraperitoneally with AG at 10, 25, and 50 mg/kg. AG (A800173, Macklin, Shanghai, China) was dissolved in DMSO and diluted in saline, administered 5 days before surgery, on the day of surgery, and on the first day after surgery. Then, the mice were randomly divided into 3 groups, namely, Sham, I/R and I/R + AG (25 mg/kg), for the TTC staining, Western blot, qRT-PCR, gelatin–ink imaging and biochemical index detection assays.

#### 4.9.3. Echocardiography

After 24 h of reperfusion, the mice were anesthetized again, and their heart function was examined with a high-resolution small-animal echocardiography system (VisualSonics, Toronto, ON, Canada). Parastemal short-axis view was used for LVEF, LVFS, and LVIDd.

#### 4.9.4. Determination of Myocardial Infarct Size

The size of the myocardial infarction was determined by TTC staining. Hearts were removed from the bodies after 24 h of reperfusion and immediately immersed in saline before being frozen at −80 °C for 10 min. Then, the hearts were cut perpendicularly to the long axis into five pieces (1–2 mm thick). The slices were put into a 1% TTC (Sigma, St. Louis, MO, USA) solution at 37 °C in the dark for 1–3 min. Viable myocardium is brick-red due to dehydrogenase activity; infarcted areas remain pale. Infarct size was measured using Image-Pro Plus software (version 6.0) and expressed as a percentage of the left ventricle that was pale (infarcted) [41].

#### 4.9.5. Histological Hematoxylin and Eosin (HE) Staining

Heart tissue was fixed for one night with 4% paraformaldehyde and then dehydrated, cleared, and embedded in paraffin according to the normal procedure. Continuous 5-micrometer sections were cut along the short axis of the left ventricle and placed on slides for HE staining. Cardiomyocyte necrosis, inflammatory cell infiltration, and interstitial edema could be seen with the naked eye.

#### 4.9.6. Western Blot Analysis

Total protein was extracted from myocardial tissues and CMECs using RIPA (P0013B, Beyotime, Shanghai, China) lysis buffer that contained PMSF (ST507, Beyotime, Shanghai, China) protease inhibitor. Protein concentration was measured through the BCA (P0012, Beyotime, Shanghai, China) method. The same quantity of proteins was separated via SDS-PAGE and then moved onto NC membranes. The membranes were blocked for 1 h at room temperature with 5% skimmed milk before being incubated overnight at 4 °C with the following primary antibodies: S100A8 (A15315, Abclonal, Wuhan, China), IL-17A (A12454, Abclonal, Wuhan, China), IL-17RA (A10052, Abclonal, Wuhan, China), Act1 (A6776, Abclonal, Wuhan, China), p-NF-κB p65 (1:500, AP0124, Abclonal, Wuhan, China), NF-κB p65 (1:500, A00284-1, Boster, Wuhan, China), β-actin (1:2000, TA-09, Zhongshan Golden Bridge, Beijing, China), and GAPDH (1:2000, TA-08, Zhongshan Golden Bridge, Beijing, China). Except otherwise specified, all the primary antibodies were diluted at 1:500. After washing with TBST, the membranes were incubated with HRP-conjugated secondary antibodies (anti-mouse, 1:2000, ZB2305; anti-rabbit, 1:2000, ZB2301; Zhongshan Golden Bridge, Beijing, China) for 1 h at room temperature. Protein bands were identified using ECL reagent (M2301, Harbin, China) and observed with an Odyssey infrared imaging system (LICORbio, Lincoln, NE, USA). Band intensity was measured using ImageJ software (version 1.54) and normalized to GAPDH or β-actin.

#### 4.9.7. Quantitative Real-Time PCR (qRT-PCR)

Total RNA was isolated from myocardial tissue with Trizol reagent (15596026, Invitrogen, Thermo Fisher Scientific, Waltham, MA, USA). Reverse transcription was carried out using a PrimeScript™ RT reagent kit (RR037A; Takara Bio Inc., Kusatsu, Shiga, Japan) to generate cDNA. Primers were made by Sangon Biotech Co., Ltd. (Shanghai, China). Primer sequences are given in Table 1. PCR amplification was performed with a SYBR Premix Ex Taq™ kit (SQ121-02, Innovagene, Hunan, China). Thermal cycling conditions: 95 °C for 30 s as an initial denaturation step, followed by 40 cycles of 95 °C for 5 s as a denaturation step, and 60 °C for 30 s as an annealing/extension step. Relative mRNA expression levels of target genes (*S100A8*, *Fak*, *Src*) were determined using the 2^−ΔΔCt^ method with β-actin as the internal reference gene.

#### 4.9.8. ELISA and LDH Activity Assay

Myocardial tissue homogenate was acquired. The protein concentrations of IL-1β, IL-6, and TNF-α were measured with commercial ELISA kits (IL-1β: RK00006, IL-6: RK00008, TNF-α: RK00027, ABclonal, Wuhan, China) as per the manufacturer’s instructions. The quantity of lactate dehydrogenase (LDH), which serves as a sign of cellular harm, was evaluated in myocardial tissues and the cell culture supernatant by means of an LDH assay kit (P0395S, Beyotime, Shanghai, China).

#### 4.9.9. Gelatin–Ink Infusion for Microvascular Visualization

Gelatin–ink staining was employed to examine the small blood vessels harmed by I/R injury. Gelatin powder (9000-70-8, Sigma, St. Louis, MO, USA) and black ink (Yidege, Beijing, China) were mixed together to get a final concentration of 3%, which was heated until fully dissolved and maintained at 37 °C. After anesthesia, the warm (37 °C) gelatin–ink solution was perfused into the external jugular vein at room temperature, in the range of 25–30 °C. When the limbs and lips of the mice turned black, their thoraxes were opened, and the great vessels at the base of the heart and the superior and inferior vena cava were tied off. And then, the mice were put into a cold room with a temperature of about 4 degrees Celsius for around 4 h to make the gelatin–ink solution cool down and harden. Then, the hearts were taken out and put into 4% paraformaldehyde for 36 h; then, the hearts were put into a sucrose solution and prepared for frozen sectioning.

#### 4.9.10. In Vitro Culture of CMECs and H/R Injury Model

CMECs were bought from CHI Scientific (Jiangyin, China). The cells were cultured in DMEM (PYG0073, Boster, Wuhan, China), containing 100 μg/mL penicillin/streptomycin (C0222, Beyotime, Shanghai, China) and 20% FBS (FB25015, Clark Bioscience, Richmond, VA, USA) in a humidified incubator at 37 °C with 5% CO_2_. When the cells reached 80% confluence, they were subcultured, and their typical cobblestone morphology was visualized under an optical microscope (Olympus, Tokyo, Japan). Cells from passages 2–4 were used in this experiment. An H/R injury model was created in vitro to imitate I/R. CMECs were cultured in a tri-gas incubator (92% N_2_, 3% O_2_, 5% CO_2_) at 37 °C for 12 h in serum-free medium under hypoxic conditions. Then, the cells were returned to normal oxygen conditions (37 °C, 5% CO_2_), and they were placed in DMEM containing 5% FBS for 6 h to serve as reperfusion [42].

#### 4.9.11. CCK-8

For the CCK-8 (C0037, Beyotime, Shanghai, China) test, all cells of different groups were prepared in advance. When the cell density reached 80%, the serum was starved for 24 h before treatment. To make a CCK-8 working solution, 10 μL of CCK-8 reagent was added to 100 μL of DMEM. Then, 200 μL of this working solution was added to every well of a 96-well plate that had cells in it, and it was left to sit at 37 °C for two hours. The OD value of each well was measured by an ELISA microplate reader at 570 nm.

#### 4.9.12. Immunofluorescence Staining

Cells were incubated with a primary antibody against S100A8 (1:300, A15315, Abclonal, Wuhan, China) overnight at 4 °C. Then, after washing, the samples were incubated with a TRITC-conjugated secondary antibody (1:50, TRITC: ZF-0313, Zhongshan Golden Bridge, Beijing, China) diluted in PBS for 1 h at room temperature in the dark. After another wash, the nuclei were stained with DAPI (C1005, Beyotime, Shanghai, China) for 10 min at room temperature in the dark. Staining results were observed and photographed on a live-cell workstation (Leica, Wetzlar, Germany).

#### 4.9.13. Overexpression DNA

S100A8 full-length plasmids inserted in pcDNA3.1 vector were synthesized by Miaoling Biotechnology (Wuhan, China). An empty vector was used as the negative control. The transfection of the overexpression plasmid was performed using Lipofectamine 2000 transfection reagent (TL201-01, Vazyme, Nanjing, China).

#### 4.9.14. Statistical Analysis

Data were analyzed with SPSS 19.0 and shown as mean ± SD. Differences among groups were analyzed using a *t*-test or one-way ANOVA according to the situation. *p* < 0.05 was considered statistically significant.

## 5. Conclusions

To put it briefly, this research has not only exposed the endothelial cell-controlled hub system during MI/R injury, but it has also developed and validated a novel “data-driven, AI-accelerated, closed-loop verification” research model. It combines single-cell dynamic atlases, virtual cells, and AI forecasts into a quick iteration cycle of panoramic atlas mapping → smart hub target extraction → rapid intervention drug screening → multi-level experimental testing. The creation of this model represents a significant shift in MI/R studies away from the traditional “hypothesis-driven, step-by-step search” approach towards a “data-driven, intelligent search” method. Its main advantage is the use of AI technology to connect high-dimensional omics information and perform causal reasoning, enabling extremely fast checks of important control points and key methods to intervene in difficult diseases. From dissecting the mechanism to finding and verifying the function of the candidate drug AG in this study, it is a story of success showing the effectiveness of this method. So, the great importance of this work is that it provides a potential solution to the big problem of creating drugs for hard-to-treat diseases, such as MI/R, which takes a long time, costs a lot of money, and often fails. Artificial intelligence can be integrated throughout the process of target discovery and drug repurposing; in this manner, it may possibly drastically cut down the length of the typical early discovery and preclinical validation procedure that normally lasts several years, thereby accelerating the development and clinical application of the next generation of exact therapies. In the future, this research paradigm will be widely used and comprehensively transform the landscape of cardiovascular drug discovery.

## Figures and Tables

**Table 1:** Primer sequences for qRT-PCR.

Gene	Sequences (5′–3′)
*Src*	Forward: CTTCCTCGTGAGGGAGAGTG
	Reverse: TGGGACACACGGTAGTGAGA
*Fak*	Forward: CTAGCCACGGTGGATGAGAC
*S100A8*	Reverse: TGCTGATGAGCTCGCCTAAG Forward: AGTGCCCTCAGTTTGTGCAG Reverse: CGCCCACCCTTATCACCAAC
*β-actin*	Forward: CGCCACCAGTTCGCCATGGA
	Reverse: TACAGCCCGGGGAGCATCGT

## Data Availability

The original contributions presented in this study are included in the article. Further inquiries can be directed to the corresponding authors.

## Abbreviations

The following abbreviations are used in this manuscript:

Act1	NF-κB activator 1
AMI	Acute myocardial infarction
AG	Andrographolide
CD14	Cluster of differentiation 14
HE	Hematoxylin and eosin
H/R	Hypoxia/reoxygenation
ICAM-1	Intercellular adhesion molecule 1
IL-8	Interleukin 8
IL-17A	Interleukin 17A
IL-17RA	Interleukin-17 receptor A
I/R	Ischemia reperfusion
KEGG	Kyoto Encyclopedia of Genes and Genomes
MCP-1	Monocyte Chemoattractant protein 1
MI/R	Myocardial ischemia reperfusion
NF-κB	Nuclear Factor-kappa B
S100A8	S100 calcium binding protein A8
TLR	Toll-Like Receptor
TTC	2,3,5-Triphenyltetrazolium chloride
VCAM-1	Vascular cell adhesion molecule 1
